# Editorial: Optimized gene-engineering and combination therapies to boost γδT cell immunotherapeutic performance

**DOI:** 10.3389/fimmu.2024.1414812

**Published:** 2024-04-18

**Authors:** Marta Barisa, Daniel Abate-Daga, Jonathan Fisher

**Affiliations:** ^1^Developmental Biology & Cancer Department, University College London Great Ormond Street Institute of Child Health, University College London, London, United Kingdom; ^2^Department of Immunology, H. Lee Moffitt Cancer Center & Research Institute, Tampa, FL, United States

**Keywords:** γδT cell, immunotherapy, oncology, CAR-T, BiTe, checkpoint blockade (ICB) therapy

This collection of original research articles, reviews and perspectives summarizes the state-of-the-art in γδT cell immunotherapy, and examines it in the broader context of allogeneic chimeric antigen receptor (CAR-T) therapies for cancer. The topics covered include a review of specific γδT cell clinical trials, alone or in the context of alternative allogeneic cellular immunotherapy approaches, and a range of pre-clinical studies that focus on γδT cell combination with checkpoint blockade, modulators of the cholesterol biosynthesis pathway, bispecific T cell engagers (BiTes), angiogenic blockers, as well as γδT cell therapeutic homing, enhanced methods of γδT cell product manufacture, and, finally, an overview of the latest in γδT cell synthetic engineering.

Interest in cancer immunotherapy using non-canonical lymphocytes has grown steadily since the early 2000s (1, 2). Much of this interest is driven by the perception of specific limitations of the more widely adopted gene-modified αβT cell therapies (3), which have produced transformative shifts in the treatment of CD19^+^ and BCMA^+^ B cell malignancies, but have yet to produce similar breakthroughs in the treatment of other leukaemia types or solid tumours. Additionally, the major histocompatibility complex (MHC) recognition-driven alloreactivity of peripheral αβT cells has restricted their use predominantly to autologous adoptive transfer, which is accompanied by high cost and complex logistics of product manufacture (4).

γδT cells, natural killer T cells (NKT) and NK cells are all alternative cytotoxic lymphocyte (CTL) sources that are MHC non-restricted and do not cause graft *versus* host disease (GvHD). All are further easily accessible in the peripheral blood of healthy donors, from which they can be expanded and genetically modified using GMP-compatible methods. γδT cells offer a particularly attractive route for cellular immunotherapy development, as their phenotype combines features of a range of the afore-mentioned cells. Like classical αβT cells as well as NKT cells, γδT cells express a T cell receptor (TCR). What defines the γδT cell subset is its expression of TCRγ/δ as opposed to TCRα/β heterodimers. While different TCRγ/δ clones have been found to engage various atypical MHCs loaded with sulfatide or lipid antigens, as well as butyrophilin and butyrophilin-like molecules, TCRγ/δ biology and ligand recognition remain poorly understood (5).

In addition to the TCR, γδT cells express a range of receptors that are also expressed by NK cells. These are activated by ligand patterns of cellular stress and transformation, and include NKG2D, DNAM-1, NKp30 and NKp44. Both NK and γδT cells can further express receptors that engage humoral immunity, including Fc receptor CD16. Upon target engagement, human γδT cells can exhibit prolific Th1-type cytokine production and cytotoxicity. Murine γδT cells further appear to present with a thymically-determined Th1/Th17 functional dichotomy characterised by IFN-γ and IL-17 production, respectively, though the degree to which this is relevant for primate γδT cell biology remains unknown (6).

Olofsson et al. open this Research Topic by exploring γδT cell anti-tumour functionality in their article on Vγ9Vδ2 cell tumour antigen cross-presentation to αβT cells. Vγ9Vδ2 cells are the most common peripheral γδT cell subset, and their ability to cross-present antigens has been described in several contexts (7, 8). This unique aspect of their biology represents a significant additional route of immune response modulation that γδT cells possess in contrast to αβT cells or NK cells.

Despite this range of features that make them attractive for cellular oncoimmunotherapy, clinical trials testing γδT cell adoptive transfer interventions have produced mixed results. Ling Ma et al. provide a comprehensive overview of the data that has been published on a range of γδT cell adoptive immunotherapy trials. Smirnov et al. then expand on this further with their review, placing γδT cell studies in the broader context of allogeneic CAR-T clinical efforts at large, where γδT cells are considered alongside TCR-knockout or otherwise modified αβT cells, virus-specific CTLs and induced pluripotent stem cells. Lv et al. continue this theme with their review, which explores current approaches to overcome allogeneic cellular immunotherapy GvHD and host rejection. Their review considers γδT cell immunotherapy alongside that of NK cells, NKT cells, mucosal invariant T cells and pluripotent stem cells.

The most sizeable portion of the Research Topic focuses on pre-clinical data reports that examine γδT cell therapeutic combinations. In all cases, the type of γδT cell discussed is the peripherally-dominant Vγ9Vδ2 subset. Liou et al. describe a novel approach to modulating TCR engagement by increasing tumour cell accumulation of the Vγ9Vδ2-TCR ligand, isopentenyl pyrophosphate (IPP). They achieved this by knocking out the IPP-catalyzing enzyme, farnesyl diphosphate synthase, using short-hairpin RNA. This work is followed by a range of studies examining Vγ9Vδ2 cell checkpoint receptor expression and blockade, with a compelling if complex set of results.

Ridgley et al. examined Vγ9Vδ2 T cell checkpoint receptor expression following phosphoantigen challenge, and found that, in the context of their THP-1 acute myeloid leukaemia model, TIM-3, LAG-3 and NKG2A, but not PD-1, were promising targets for checkpoint blockade. Curiously, however, they reported that – despite the substantial upregulation of these receptors upon T cell challenge – the team were unable to identify a cytotoxic or cytokine benefit of applying checkpoint blockers, speculating instead that these may play a more important role in de-repressing T cell proliferation. This was in some contrast to a report by Lui et al., where PD-1 blockade was efficacious at enhancing Vγ9Vδ2 cell immunotherapy against mesothelioma *in vitro* and *in vivo*, especially against PD-L1 high tumours, but not in a manner that was dependent on pyroptosis. Giannotta et al., meanwhile, reported that, in the context of multiple myeloma, PD-1^+^ bone marrow Vγ9Vδ2 T cells exhibited phenotypic, functional alterations that are consistent with chronic exhaustion and immune senescence. Importantly, they found that PD-1, TIM-3 and LAG-3 checkpoints were upregulated on Vγ9Vδ2 cells in a hierarchical manner, and that the blockade of specific combinations of these could exacerbate, rather than rescue, γδT cell dysfunction. Their data indicated that a PD-1/LAG-3 blockade combination is the most effective in the context of multiple myeloma. The group concluded that immune checkpoint blockade should be tailored to the disease to enhance the positive and minimise the negative effects - an observation that is likely relevant for all γδT cell therapeutic combinations.

Yang et al. evaluated Vγ9Vδ2 cell checkpoint interactions in the context of targeting with BiTes, specifically anti-PD-L1 x anti-CD3 BiTes. A therapeutic combination of expanded Vγ9Vδ2 cells with BiTe was efficacious against models of PD-L1-expressing non-small cell lung carcinoma. Branella et al. took an alternative approach to Vγ9Vδ2 cell BiTes. The group developed an acute myeloid leukaemia-targeting CAR-γδT cell that also secreted a BiTe against c-kit, both knocked in via transient transfection. The CAR/BiTe-modified γδT cells moderately extended survival of NSG mice engrafted with disseminated AML, but therapeutic efficacy was limited by a lack of γδT cell homing to murine bone marrow. This report was followed by a second report from the same group (Trent Spencer, Emory), where Parwani et al. examined the lack of Vγ9Vδ2 cell homing to NSG mouse bone marrow in greater detail. Interestingly, while they showed that total body irradiation of the animals could increase human γδT cell migration to the bone marrow, this was passive accumulation rather than homing. γδT cell homing could be induced by providing sources of CCL-2 within the tumour microenvironment.

Bold et al. reported a new way to manufacture Vγ9Vδ2 cells in a GMP-compatible manner, by switching from RPMI1640-based media to CTS OpTmizer-based media, and increasing both zoledronic acid and IL-2 concentrations, as well as extending expansion period, in order to achieve greater cytotoxic efficacy of their products.

Zhang et al. reported an unexpected finding in murine models of breast cancer, whereby low-dose VEGFR2 mAb or VEGFR2-tyrosine kinase inhibitors were efficacious, while high-dose VEGFR2 mAb was not. The mechanism they identified for this was that high-dose anti-VEGFR2 mAb treatment elicited IL-17A expression in resident γδT cells via VEGFR1-PI3K-AKT pathway activation, and that this then promoted N2-like neutrophil polarization and consequent shaping of the tumour microenvironment to a suppressive state. While compelling, given the species differences, it remains unclear how directly this applies to human γδT cells and breast cancer.

To conclude the Research Topic, Yuan et al. summarize and critically evaluate the latest developments in γδT cell synthetic engineering, covering topics like CAR-T, TCR gene transfer and combination with γδT cell engagers. The team then discuss the implications of these latest engineering strategies, and the challenges that lie ahead for engineered γδT cell monotherapy and combinatorial approaches. As this collection of articles highlights, much exciting pre-clinical and clinical exploration of γδT cell combinatorial and gene-modified approaches is taking place. The coming decade of clinical trial data will shape the direction of the γδT cell immunotherapy field within oncology and beyond.

## Author contributions

MB: Writing – original draft, Writing – review & editing. DA-D: Writing – review & editing. JF: Writing – review & editing.
